# Superior Anti-arrhythmogenic Effect of Combined Conditioning with Nicotinamide Mononucleotide and Ubiquinol in Myocardial Ischemia/Reperfusion Injury in Aged Rats

**DOI:** 10.34172/apb.2024.044

**Published:** 2024-04-15

**Authors:** Behnaz Mokhtari, Amirhossein Jessri, Samad Ghaffari, Reza Badalzadeh

**Affiliations:** ^1^Aging Research Institute, Tabriz University of Medical Sciences, Tabriz, Iran.; ^2^Student Research Committee, Tabriz University of Medical Sciences, Tabriz, Iran.; ^3^Cardiovascular Research Center, Tabriz University of Medical Sciences, Tabriz, Iran.; ^4^Molecular Medicine Research Center, Tabriz University of Medical Sciences, Tabriz, Iran.; ^5^Department of Physiology, Faculty of Medicine, Tabriz University of Medical Sciences, Tabriz, Iran.

**Keywords:** Aging, Arrhythmia, Cardioprotection, Myocardial reperfusion injury, Nicotinamide mononucleotide, Ubiquinol

## Abstract

**Purpose:**

Lethal ventricular arrhythmias are a significant clinical concern following reperfusion therapies in elderly patients with myocardial infarction. The combination of multi-target therapies to achieve optimal anti-arrhythmogenesis and improve the chances of successful translation for patient benefit has prompted considerable interest. This study examined the anti-arrhythmic effect of nicotinamide mononucleotide (NMN)/ubiquinol combination treatment following myocardial ischemia/reperfusion (IR) injury in aged rats, with an emphasis on the role of oxidative stress and nitric oxide (NO).

**Methods:**

Male Wistar rats (n=30, 22-24 months old, 400-450 g) were randomized into five groups with or without IR and/or NMN and ubiquinol, either alone or in combination. NMN (100 mg/kg/48 hours) was administered intraperitoneally for 28 days before IR, and ubiquinol (30 mg/kg) was injected intravenously at early reperfusion. Electrocardiographic signals were recorded during the ischemia and the first 30 minutes of reperfusion. Two hours after reperfusion, myocardial hemodynamic and LDH release were measured, and the left ventricle samples were obtained to evaluate oxidative stress markers and NO levels.

**Results:**

NMN/ubiquinol combination treatment significantly minimized the occurrence and severity of IR-induced arrhythmias, improved myocardial function, and reduced LDH release (*P*<0.05). It also decreased MDA content, increased superoxide dismutase (SOD), glutathione peroxidase (GP_X_), and catalase (CAT) activities, and enhanced NO formation (*P*<0.05). This combined treatment showed greater efficacy than the single treatments.

**Conclusion:**

This study revealed the anti-arrhythmic effect of NMN/ubiquinol combination treatment in IR-treated aged rats, which may be associated with reduced oxidative stress and increased NO formation. This combinational approach deserves more investigation due to its potential to confer better anti-arrhythmic effect during aging.

## Introduction

 Effective management of acute myocardial infarction (AMI) is based on pharmacological or interventional reperfusion that restores the blood supply to the ischemic myocardium; however, reperfusion therapy causes myocardial ischemia/reperfusion (IR) injury, especially fatal ventricular arrhythmias.^1^ A major culprit for the development of reperfusion arrhythmogenesis is oxidative stress, which serves as an upstream signal for altering ion homeostasis and gap junction remodeling. A rapid burst of reactive oxygen species (ROS) in the heart triggers acute arrhythmias, while long-term post-translational alterations of oxidative stress-related proteins and changes in genotypes are associated with chronic arrhythmias.^2,3^ Studies have shown that oxidative stress during reperfusion promotes superoxide-induced nitric oxide (NO) inactivation, an arrhythmias-promoting event that leads to excessive formation of oxygen-derived free radicals during IR injury.^4^ Thus, electrophysiological remodeling and arrhythmias that occur after myocardial IR could be a direct result of oxidative stress and reduced NO bioavailability.^5,6^

 Minimizing IR-induced arrhythmias and restoring normal electromechanical activity of the heart are of great clinical importance. However, the success of pharmacological and mechanical efforts validated in animal-based studies to minimize the adverse arrhythmogenic events associated with myocardial IR injury has been disappointing in patient-based studies.^7-9^ The failure in the translational process has been attributed to suboptimal animal studies that are far from the clinical situation, particularly due to the neglect of co-medications, co-morbidities, and risk factors such as aging.^10^ Aging develops a plethora of molecular changes that decrease the tolerance and survival of cardiomyocytes following the IR insult, aggravate the occurrence of arrhythmias, and confound the anti-arrhythmogenic efficacy of therapeutic strategies.^11^ Another reason for the translational gap is that many of experimental studies applied just one cardioprotective intervention focused on a specific signaling pathway or target within the cardiomyocyte, an approach that has suboptimal and sometimes inconsistent effects in clinical setting.^12^ As such, successful translation of cardioprotection against IR-induced arrhythmias may require more research conducted in animal models of translational value, where co-medications, co-morbidities, and risk factors are taken into consideration, as well as the development of multi-target therapeutic approaches directed to multiple end-effectors and signaling pathways.^13,14^

 Nicotinamide mononucleotide (NMN), an intermediate of nicotinamide adenine dinucleotide (NAD^+^) biosynthesis, possesses anti-aging activity and has critical role in a wide range of biological processes of the body including deoxyribonucleic acid repair, energy homeostasis, gene expression, aging, and cell death.^15,16^ Considering the wide range of altered mitochondrial mechanisms upon myocardial IR injury and aging, single application of therapeutic agents aiming to promote mitoprotection may be ineffectual.^9,17^ Emerging data suggests that combining NMN with some agents can be considered as an effective therapeutic regiment to produce better efficacy and create sufficient cardioprotection during aging.^18,19^ Ubiquinol, the active form of coenzyme Q_10_, exerts key functions in mitochondrial electron transport chain and scavenging ROS, promoting the regeneration of antioxidants, and protecting cell membranes from lipid peroxidation.^20,21^ Upon aging as well as myocardial IR injury, the contents of NMN and ubiquinol in the heart are remarkably declined, leading to oxidative stress and progressive impairment of mitochondrial function.^18^ Hence, considering the idea of NMN/ubiquinol combination therapy may be of great importance to pursue in order to minimize the adverse arrhythmogenic events associated with myocardial IR injury.

 Given the therapeutic potentials of NMN and ubiquinol, we decided to examine the effectiveness of their combined treatment in mitigating IR-induced arrhythmias. To somewhat narrow the gap between experimental and clinical studies, we felt the need to develop an aged rat model and use NMN and ubiquinol in a combination mode to evaluate whether this therapeutic approach can provide better efficacy and is potent enough to minimize the adverse arrhythmic events related to myocardial IR injury upon aging.

## Materials and Methods

###  Experimental animals 

 The experiments were conducted on male Wistar rats (n = 30, 22-24 months of age, weighing 400–450 g) that were provided by the animal care unit of Tabriz University of Medical Sciences. Rats were allocated to standard cages (three rats in each cage) and transferred to a specific pathogen-free animal breeding room with controlled temperature (25 ± 2 °C) and humidity (55 ± 10%), under a 12:12 hours light/dark schedule. They were offered unrestricted access to water and standard laboratory rodent chow. All rats were allowed to adapt the environment for 2 weeks. Animal handling and experimental procedures were carried out under the supervision of the Ethics Board of Tabriz University of Medical Sciences (Permission numbers: IR.TBZMED.VCR.REC.1399.283, and IR.TBZMED.VCR.REC.1399.202), following the recommendations in the Guide for the Care and Use of Laboratory Animals published by the United States National Institutes of Health (8th Edition, National Research Council, 2011).

###  Grouping and administration

 Five groups, each comprising 6 aged rats, were formed through a random assignment process:

- Group I (Sham): Untreated rats were subjected to open-chest surgery without left anterior descending coronary artery (LAD) occlusion and re-opening;
- Group II (IR): Untreated rats were subjected to myocardial IR injury modeling;
- Group III (IR + NMN): Rats received intraperitoneal injections of 100 mg/kg/48hours NMN (Sigma-Aldrich, USA) dissolved in normal saline for 4 weeks prior to myocardial IR injury modeling;^18^
- Group IV (IR + Ubi): IR-treated rats received an intravenous injection of 30 mg/kg ubiquinol (Sigma-Aldrich, USA) at early reperfusion;^18^ and
- Group V (IR + NMN + Ubi): IR-treated rats received both NMN and ubiquinol as in groups III and IV.

Electrocardiographic signals were continuously recorded during the ischemia and the first 30 minutes of reperfusion. In the sham group, the electrocardiographic signals were monitored for a duration matching that of the ischemia and reperfusion phases in the IR group. Hemodynamic measurements were conducted two hours after either the sham surgery or the IR procedure. After obtaining cardiac blood sample and excising the heart under deep anesthesia, the animals were euthanized. Subsequently, heart and serum samples were preserved at −80 °C for subsequent analyses.

###  Construction of myocardial IR injury

 An *in vivo* model of myocardial IR injury was established using previously described method.^22^ In brief, rats received an intraperitoneal injection of ketamine/xylazine (60:10 mg/kg) for anesthesia. They were then endotracheally intubated and ventilated with room air (tidal volume: 2–3 ml/kg, respiratory rate: 65–70/min, ratio of exhalation/inhalation: 1:1). To expose the heart, a left thoracic incision was made in the fourth intercostal space. Then, myocardial ischemia was set up by ligating the LAD close to its origin using a 6–0 silk suture. Criteria for successful occlusion were the appearance of epicardial cyanosis and ST segment elevation in lead II. The appearance of epicardial cyanosis, indicating a bluish discoloration of the heart tissue, serves as a visual marker of reduced blood flow and oxygenation during the ischemic phase. Additionally, ST segment elevation in lead II on the electrocardiogram provides an objective measure of myocardial injury and ischemic insult, further confirming the occlusion of coronary blood flow. To allow reperfusion after 30 minutes of ischemia, the ligation was removed. Subsequently, the chest was closed and sutured. The reperfusion phase lasted for 2 hours. The sham group underwent the aforementioned procedure in the absence of LAD occlusion.

###  Assessment of ventricular arrhythmias

 Continuous monitoring and recording of electrocardiographic signals were carried out during both the ischemic and the first 30 minutes of reperfusion phases using three needle electrodes attached subcutaneously. For the sham group, the electrocardiographic signals were recorded over a period of time equivalent to the duration of ischemia and reperfusion in the IR group, despite the fact that the sham group did not undergo ischemia and reperfusion procedures. A data acquisition system (PowerLab, ADInstruments, Australia) and LabChart Software (LabChart 7.3, ADInstruments, Australia) were employed to record, digitize, and analyze the arrhythmias. The classification of ventricular arrhythmias according to the Lambeth Conventions is outlined below^23^:

- Premature ventricular complexes) PVC) as a collection of single ventricular premature beats (VPB), ventricular bigeminy (VB), and ventricular salvos (VS);
- Ventricular tachycardia (VT) as the occurrence of four or more consecutive VPBs with corresponding effective left ventricular pressure; and
- Ventricular fibrillation (VF) as the irregular morphology of ventricular electrocardiograms without corresponding effective left ventricular pressure.

Analysis of electrocardiograms involved evaluating the number and duration of PVC, VT, and VF, as well as determining the severity (or score) of arrhythmias. According to the five-degree assessment system, the severity or score of arrhythmias included: 4 = VF; 3 = VT; 2 = VB and/or VS; 1 = VPB; and 0 = no arrhythmia. It is important to mention that if multiple types of arrhythmias were recorded in a single sample, it indicated the maximum degree of arrhythmia.

###  Assessment of cardiac hemodynamic changes 

 Two hours after either the sham surgery or the IR procedure, the rats underwent invasive arterial monitoring by catheterizing the right common carotid artery under deep anesthesia. The PE50 catheter was connected to the pressure amplifier and PowerLab System (ADInstruments, Australia) at one end. The opposite end of the catheter was then meticulously inserted into the left ventricle to facilitate the measurement of left ventricular functional parameters, including left ventricular end-diastolic pressure (LVEDP, in mm Hg), left ventricular developed pressure (LVDP in mm Hg), maximal ascending rate of left ventricular pressure ( + dp/dt, in mm Hg/s), and maximal descending rate of left ventricular pressure (-dp/dt, in mm Hg/s). LVDP was determined by calculating the difference between LVEDP and left ventricular systolic pressure (LVSP). The data were processed using LabChart Software (LabChart 7.3, ADInstruments, Australia).

###  Measurement of LDH level

 After completion of the hemodynamic measurements, cardiac blood samples were acquired and placed in a non-anticoagulant tube for 2 hours at 25 °C. Subsequently, the sera were separated using a centrifuge at 3000 × g for 10 minutes. To quantify myocardial damage, the enzyme activity of LDH in the sera was determined using an LDH assay kit (Parsazmoon Co., Iran) under the recommendations given by the manufacturer. The absorbance of the samples was detected spectrophotometrically at a wavelength of 492 nm. Values were expressed in International Unit per liter (IU/L).

###  Measurement of oxidative stress markers 

 After completing the hemodynamic measurements and obtaining cardiac blood samples under deep anesthesia, the animals were euthanized following the excision of their hearts. Subsequently, the LAD territory in each sample was quickly collected, homogenized, and centrifuged to obtain the supernatant. Specific kits (ZellBio GmbH, Ulm, Germany) were used to detect the content of malondialdehyde (MDA) and the activities of superoxide dismutase (SOD), glutathione peroxidase (GP_X_), and catalase (CAT), following the manufacturer’s instructions. The MDA content was reported in nmol/mg of protein, while the results for SOD, GPx, and CAT activities were presented in IU/mg of protein.

###  Determination of NO level

 The concentration of NO was determined in the supernatant samples taken from the LAD territory homogenates by using Griess colorimetric method following the provided protocols of the NO assay kit (Cib Biotech Company, Iran). This assay was based on measuring stable metabolites of NO in the supernatant after enzymatic reduction of nitrate to nitrite by vanadium (III) chloride, which was followed by color development with Griess reagent. To calibrate this assay, the standard solutions of sodium nitrite was employed. The absorbance was measured spectrophotometrically at a wavelength of 540 nm, and the values were expressed as µmol/mg of protein.

###  Statistical analysis 

 Values were reported as mean ± standard deviation. The nonparametric Kruskal–Wallis test was applied for analyzing the results for the number and duration of arrhythmias among the groups. For analyzing the remaining parameters, one-way analysis of variance (ANOVA) followed by Tukey *post hoc* test was used. The limit of statistical significance was set at a value less than 0.05 (*P*< 0.05).

## Results and Discussion

###  Effects of NMN and/or ubiquinol on the number, duration, and severity of arrhythmias

**Figure 1:**
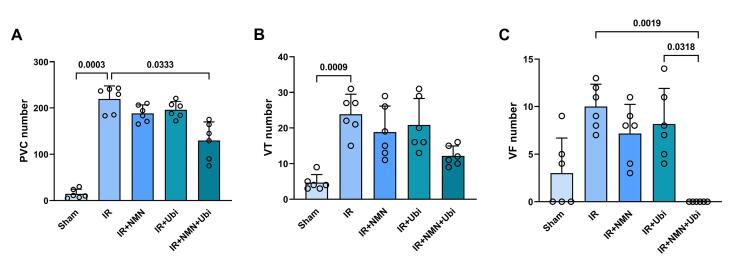
** Combination treatment reduced the number of arrhythmias following myocardial IR injury in aged rats.** The number of premature ventricular complexes (PVC) **(A)**, ventricular tachycardia (VT) **(B)**, and ventricular fibrillation (VF) **(C)** in experimental groups. The data were presented as mean ± standard deviation (n = 6/group). IR: ischemia/reperfusion; NMN: nicotinamide mononucleotide; Ubi: ubiquinol

**Figure 2:**
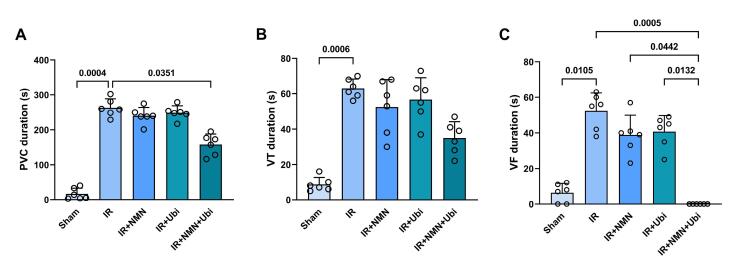
** Combination treatment reduced the duration of arrhythmias following myocardial IR injury in aged rats.** The duration of premature ventricular complexes (PVC) **(A)**, ventricular tachycardia (VT) **(B)**, and ventricular fibrillation (VF) **(C)** in experimental groups. The data were presented as mean ± standard deviation (n = 6/group). IR: ischemia/reperfusion; NMN: nicotinamide mononucleotide; Ubi: ubiquinol

**Figure 3:**
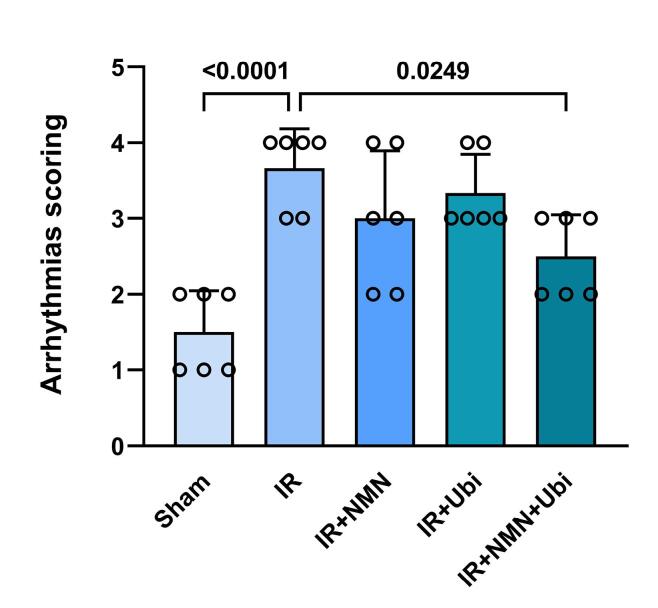
** Combination treatment reduced the severity of arrhythmias following myocardial IR injury in aged rats.** The data were presented as mean ± standard deviation (n = 6/group). IR: ischemia/reperfusion; NMN: nicotinamide mononucleotide; Ubi: ubiquinol

 According to Figure 1A and B, the IR group had a higher number of PVC and VT compared to the Sham group (*P =*0.0003 and *P =*0.0009,respectively). Although the number of PVC, VT, and VF in the NMN-pretreated group or ubiquinol-treated group was lower than the IR group, statistical analysis did not reveal any significance in these changes (Figure 1A-C). When aged rats received combined conditioning, the number of PVC and VF was further decreased compared to the IR group (*P*= 0.0333 and *P =*0.0019,respectively) (Figure 1A and C), and that the number of VF was completely diminished and reached to zero (Figure 1C). Furthermore, combined conditioning resulted in a significant decrease in the number of VF compared to the single use of ubiquinol (*P =*0.0318) (Figure 1C). Following induction of myocardial IR injury in aged rats, the durationof PVC, VT, and VF was significantly increased compared to the Sham group (*P =*0.0004, *P =*0.0006,and* P =*0.0105,respectively) (Figure 2A-C). The durationof PVC, VT, and VF was slightly but not significantly decreased following alone application of NMN or ubiquinol compared to the IR group (Figure 2A-C). When both therapeutics were administered simultaneously, the durationof PVC and VF was significantly reduced compared to the IR group (*P =*0.0351and* P =*0.0005,respectively) (Figure 2A and C), and that the durationof VF was completely diminished and reached to zero (Figure 2C). Moreover, combined conditioning resulted in a significant decrease in the durationof VF compared to the single use of NMN (*P =*0.0442) or ubiquinol (*P =*0.0132) (Figure 2C). The severity of arrhythmias was increased following induction of myocardial IR injury in aged rats (*P <*0.0001vs. Sham group) (Figure 3). The single use of NMN or ubiquinol resulted in a slight decrease in the severity of arrhythmias comparing to that of the untreated IR group, but the difference was not statistically significant (Figure 3). Nevertheless, a significant decrease in the severity of arrhythmias was found only under combination therapy (*P =*0.0249vs. IR group) (Figure 3).

 Monotherapies, while offering some benefits, have shown limitations in effectively addressing reperfusion-induced arrhythmias in the presence of cardiovascular risk factors and co-morbidities.^24^ The complex pathophysiology of reperfusion-induced arrhythmias involves multiple interconnected pathways, including altered ion channel function, calcium handling abnormalities, oxidative stress, and inflammation.^25^ Targeting only one of these pathways with a single therapeutic agent may not be sufficient to comprehensively address the multifactorial nature of reperfusion-induced arrhythmias. The failure of monotherapies to adequately combat reperfusion-induced arrhythmias highlights the importance of developing and implementing combination therapies that can provide more comprehensive and synergistic effects.^12,26^ Combining different therapeutic modalities that target multiple pathways involved in the pathophysiology of reperfusion-induced arrhythmias simultaneously can provide additive or even synergistic benefits compared to monotherapy. This synergism can enhance the overall efficacy of treatment and provide more comprehensive protection against reperfusion-induced arrhythmias. Furthermore, combination therapies that demonstrate efficacy in animal studies may have a higher likelihood of successful translation into clinical practice due to their potential to address the complex nature of reperfusion-induced arrhythmias. This translational potential makes combination therapies an attractive option for advancing treatment options in the field of cardiovascular medicine.^12,24,27^ In the present study, monotherapy with either NMN or ubiquinol did not demonstrate superior anti-arrhythmic effects. Despite the potential benefits of NMN in improving mitochondrial function and cellular energy metabolism,^15,16^ and the critical role of ubiquinol in the electron transport chain and adenosine triphosphate (ATP) production in mitochondria,^21,28^ our findings suggest that these monotherapies may not be sufficient to effectively address the complex pathophysiology of reperfusion-induced arrhythmias. The lack of superior anti-arrhythmic effects with NMN or ubiquinol monotherapy in our study highlights the limitations of targeting single pathways or mechanisms in the treatment of reperfusion-induced arrhythmias. However, the combination therapy of NMN and ubiquinol offered significant anti-arrhythmic effects in aged rats compared to monotherapy with either of them. These findings suggest that the combined use of NMN and ubiquinol reinforces parallel or distinct protective pathways simultaneously, leading to a more effective anti-arrhythmogenic approach.

###  Effects of NMN and/or ubiquinol on cardiac hemodynamic changes and serum level of LDH

**Figure 4:**
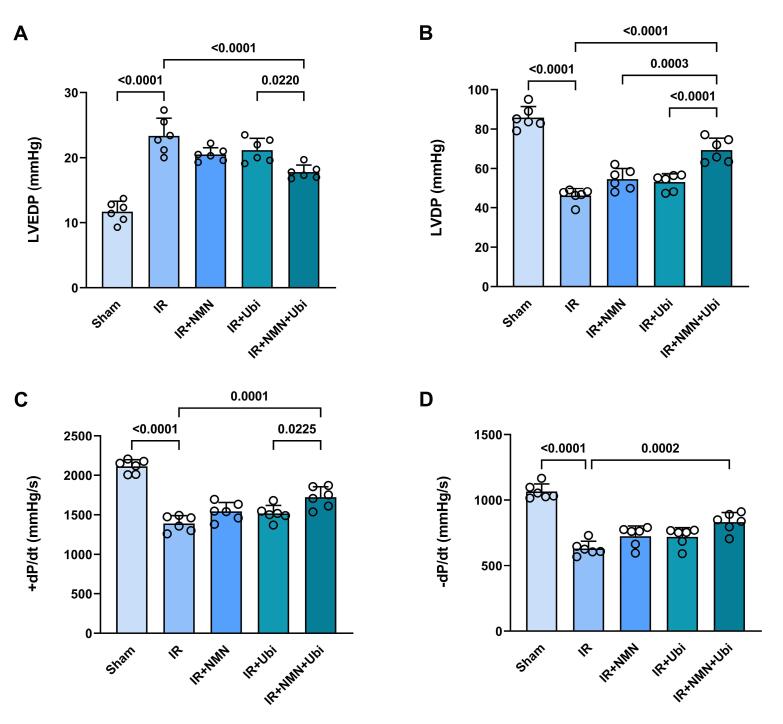
** Combination treatment improved hemodynamic parameters following myocardial IR injury in aged rats.** Left ventricular end-diastolic pressure (LVEDP) **(A)**, left ventricular developed pressure (LVDP) **(B)**, maximal ascending rate of left ventricular pressure ( + dp/dt) **(C)**, and maximal descending rate of left ventricular pressure (–dp/dt) **(D)** in experimental groups. The data were presented as mean ± standard deviation (n = 6/group). IR: ischemia/reperfusion; NMN: nicotinamide mononucleotide; Ubi: ubiquinol

**Figure 5:**
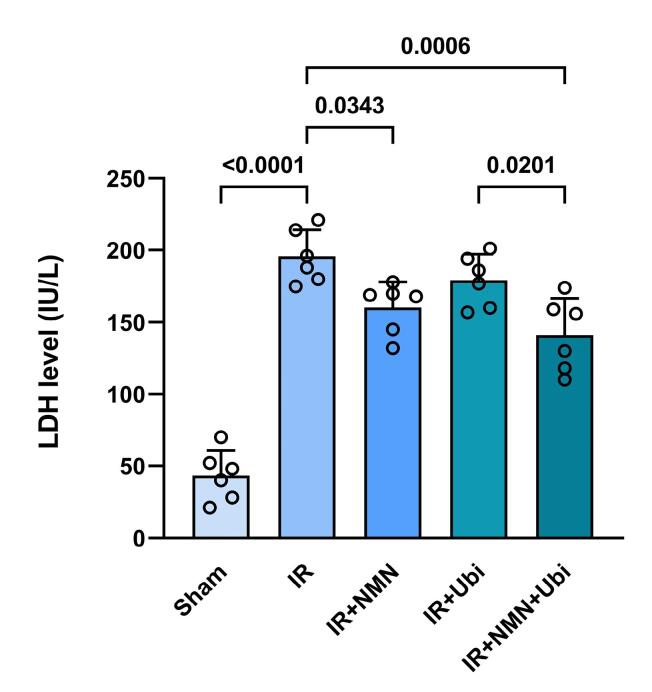
** Combination treatment reduced serum level of lactate dehydrogenase (LDH) following myocardial IR injury in aged rats.** The data were presented as mean ± standard deviation (n = 6/group). IR: ischemia/reperfusion; NMN: nicotinamide mononucleotide; Ubi: ubiquinol

 The results of the hemodynamic assessment revealed that in the experimental group exposed to myocardial IR injury, LVEDP was significantly increased (*P <*0.0001), while LVDP, *+ dp /dt*, and *– dp /dt* were significantly decreased (*P <*0.0001 for all) compared to the Sham group (Figure 4A-D). The single use of NMN or ubiquinol did not result in significant changes in the hemodynamic parameters compared to the IR group (Figure 4A-D). In contrast, the group receiving dual treatment had a different pattern of hemodynamic changes compared to the IR group, including a significant decrease in LVEDP (*P <*0.0001) and significant increases in LVDP (*P <*0.0001), *+ dp /dt* (*P =*0.0001), and *– dp /dt* (*P =*0.0002) (Figure 4A-D). Additionally, dual treatment resulted in a significant decrease in LVEDP and significant increases in LVDP and *+ dp /dt* compared to the single use of ubiquinol (*P =*0.0220, *P <*0.0001, and *P =*0.0225, respectively) (Figure 4A-C). There was also a significant difference in LVDP between the group receiving combined treatment and the group pretreated with NMN (*P =*0.0003) (Figure 4B). Figure 5 shows that following induction of myocardial IR injury, aged rats had significantly a higher level of LDH than those in the sham group (*P <*0.0001). The group that pretreated with NMN exhibited a significant reduction in LDH level compared to the untreated IR group (*P =*0.0343). Treatment of aged rats with ubiquinol did not significantly alter the level of LDH compared to the IR group. Notably, dual treatment more potently reduced LDH level compared to both the untreated IR group (*P =*0.0006) and the ubiquinol-receiving group (*P =*0.0201) (Figure 5).

 Previous studies conducted on young animal models of myocardial IR injury have demonstrated the positive effects of monotherapy with NMN or ubiquinol in mitigating the detrimental effects of IR injury.^29,30^ Conversely, findings from the present study showed that preconditioning with NMN alone or postconditioning with ubiquinol alone could not significantly improve myocardial function and reduce LDH level following IR insult in aged rats. This observation can be attributed to the multifactorial nature of age-related changes occurring in the heart, which minimizes or eliminates the efficacy of monotherapies with NMN or ubiquinol in providing cardioprotection during aging. The process of aging can adversely affect the protective pathways and prevent monotherapies from activating them. For example, aging is accompanied by a decline in mitochondrial function, including reduced ATP production, increased oxidative stress, and impaired respiratory chain activity, which not only renders the myocardium more vulnerable to injury induced by IR but also impedes the cardioprotective effects of monotherapies with NMN or ubiquinol.^31^ To address the diminished effectiveness of monotherapies with NMN or ubiquinol in the context of aging, considering combination therapy may offer a more promising alternative. The European Society of Cardiology Working Group also emphasizes that the concurrent application of therapeutic modalities directed at distinct time points during ischemia and reperfusion, and acting on the same or multiple signaling pathways, is one of the best alternatives to induce robust cardioprotection compared to their individual applications.^12^ In this regard, we deemed it ideal to use cardioprotective agents in a combination mode at distinct time points as preconditioning and postconditioning. Interestingly, we found that the application of NMN and ubiquinol in a dual manner augmented their individual cardioprotective effects in aged rats, as evidenced by improved heart function and decreased LDH release. These findings imply that aging interferes with the cardioprotective effects of monotherapies; nevertheless, combining NMN preconditioning with ubiquinol postconditioning potentiated their individual effects to neutralize the negative effects of aging on cardioprotection by either NMN or ubiquinol. Yet, further investigation is required to ascertain the mechanisms responsible for the enhanced cardioprotective effect of NMN/ubiquinol combination therapy against myocardial IR injury in aged rats.

###  Effects of NMN and/or ubiquinol on the levels of oxidative stress markers and NO formation

**Figure 6:**
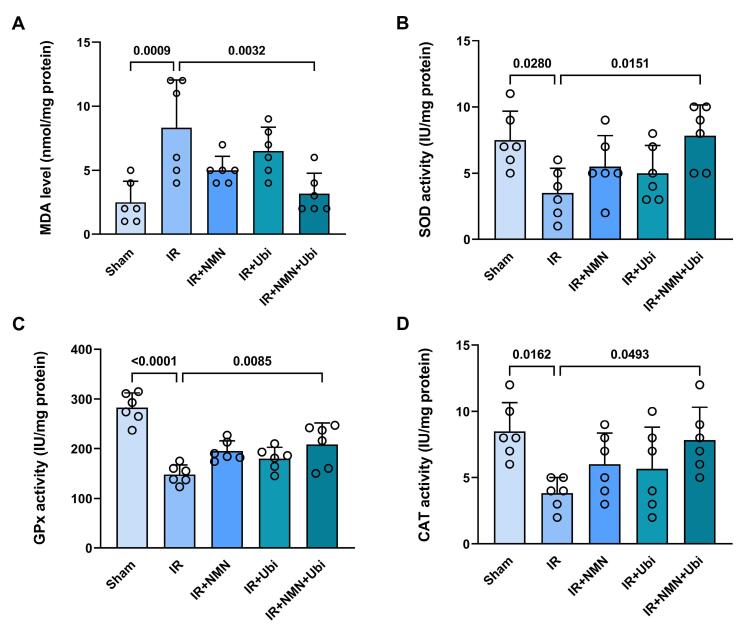
** Combination treatment reduced myocardial oxidative stress following IR injury in aged rats.** The levels of malondialdehyde (MDA) **(A)**, superoxide dismutase (SOD) **(B)**, glutathione peroxidase (GP_X_) **(C)**, and catalase (CAT) **(D)** in experimental groups. The data were presented as mean ± standard deviation (n = 6/group). IR: ischemia/reperfusion; NMN: nicotinamide mononucleotide; Ubi: ubiquinol

**Figure 7:**
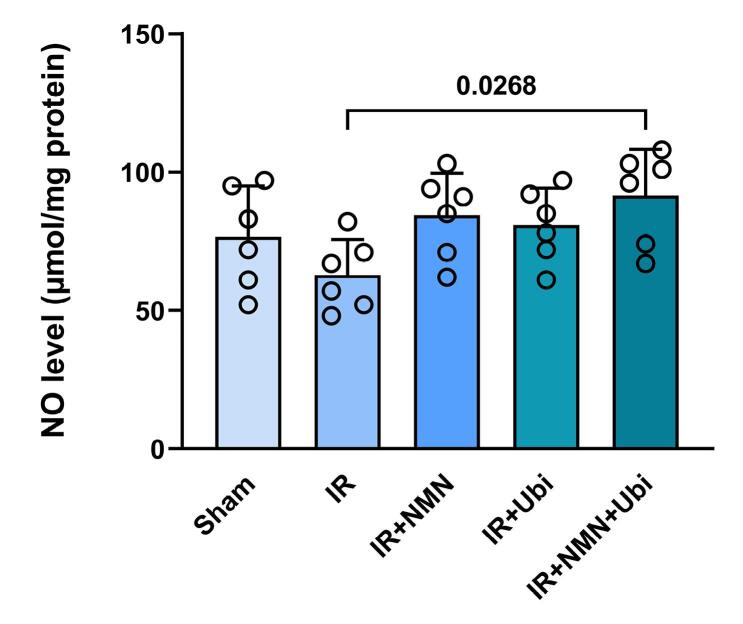
** Combination treatment reduced myocardial level of nitric oxide (NO) following IR injury in aged rats.** The data were presented as mean ± standard deviation (n = 6/group). IR: ischemia/reperfusion; NMN: nicotinamide mononucleotide; Ubi: ubiquinol

 Following induction of myocardial IR injury in aged rats, the level of MDA was significantly increased (*P* = 0.0009) (Figure 6A), whereas the activities of SOD, GPx, and CAT were significantly decreased (*P* = 0.0280, *P* < 0.0001, and *P* = 0.0162, respectively) compared to the Sham group (Figure 6B-D). The single use of NMN or ubiquinol failed to reverse IR-induced changes in MDA level and SOD, GPx, and CAT activities (Figure 6A-D). Conversely, administration of dual therapy exhibited superior efficacy in reducing MDA level (*P* = 0.0032) and increasing SOD, GPx, and CAT activities (*P* = 0.0151, *P* = 0.0085, and *P* = 0.0493, respectively) as compared to the untreated IR group (Figure 6A-D). As shown in Figure 7, the NO level was reduced to some extent in the myocardial IR-experienced group compared to the Sham group. The single use of NMN or ubiquinol had no considerable effect on the NO level. The only significant increase in the NO level was achieved following the combination of both NMN and ubiquinol (*P* = 0.0268 vs. IR group) (Figure 7).

 Previous evidence suggests that targeting NO is a possible therapeutic approach for the treatment of arrhythmias; however, the role of NO in myocardial IR injury remains controversial.^6,32,33^ Endogenous NO formation plays a protective role in preventing IR-induced arrhythmogenesis. Nevertheless, excessive formation of NO, along with increased oxidative stress, may be an important contributor to the development of arrhythmias.^32^ IR-induced oxidative stress facilitates the ventricular arrhythmias via an early afterdepolarization mechanism, promoting myocardial fibrosis, impairing gap junction conduction, and altering numerous ionic currents within the cardiomyocytes and key components of the extracellular matrix. However, there is still a lack of comprehensive understanding of molecular mechanisms by which oxidative stress exerts those effects.^4,34^ On the other hand, aging enhances oxidative stress and reduces NO bioavailability in cardiomyocytes, leading to an increase in the threshold necessary to trigger cardioprotection.^31,35^ The findings of this study indicated that the single use of NMN or ubiquinol had limited positive impacts on the levels of oxidative stress markers and NO formation in aged hearts following the IR insult, suggesting that aging diminishes the effectiveness of monotherapies. However, combined conditioning with NMN and ubiquinol effectively reduced MDA content, increased SOD, GPx, and CAT activities, and enhanced NO formation in aged IR hearts. Accordingly, it seems that the age-related loss of cardioprotection in the aged IR myocardium was overcome by the combined therapy of NMN and ubiquinol through their additive effects on reducing oxidative stress and enhancing NO formation. NMN supports the production of NAD^+^ and enhances the antioxidant defense mechanisms,^36^ while ubiquinol helps preserve mitochondrial function and neutralize ROS.^21,28^ Together, they create a potent defense mechanism against myocardial IR injury by simultaneously reducing oxidative stress and enhancing NO formation. Generally, the combined action of NMN and ubiquinol offers a comprehensive approach for neutralizing the negative effects of aging on anti-arrhythmogenic mechanisms. The mechanisms underlying this approach appear to involve the modulation of oxidative stress and NO formation pathways. Further research is required to clarify multiple upstream pathways of these findings.

## Limitations

 In this study, we employed a 2-hour reperfusion period to evaluate the anti-arrhythmic effects of the combination treatment. While this timeframe allowed us to capture immediate post-ischemic responses and early reperfusion effects, it may not fully encompass the outcomes observed during a longer reperfusion period. Extending the reperfusion duration to align with recommended criteria for acute phase assessments could offer a more comprehensive understanding of the positive effects of the combination treatment. In addition to considering the acute phase of IR injury, future studies should also evaluate the effects of NMN and ubiquinol combination therapy during the chronic phase of IR injury. Histological analyses to assess structural changes, fibrosis, and cellular responses in the heart during the chronic phase of IR injury can offer insights into the treatment’s ability to attenuate chronic cardiac remodeling and promote tissue repair following the initial ischemic insult. Moreover, we did not investigate the effects of our interventions on female rats, which limits the generalizability of the findings and may overlook important sex-related differences in cardiac responses to NMN and ubiquinol combination therapy. Additional studies should address the discrepancy in sex representation by including both male and female animals in the experimental design. Furthermore, subsequent research efforts should aim to elucidate the impact of NMN and ubiquinol on specific molecular pathways involved in arrhythmia generation, such as ion channel function, calcium handling, connexin-43 expression and intercellular communication, and inflammatory signaling.

## Conclusion

 In conclusion, this study presented that combined conditioning with NMN and ubiquinol potentiated the anti-arrhythmic effects by either NMN or ubiquinol in aged rats experiencing myocardial IR injury. The combination of NMN and ubiquinol enhanced their effects to neutralize the negative effects of aging on anti-arrhythmogenesis, achieved in part by reducing oxidative stress and increasing NO formation in aged IR myocardium. NMN/ubiquinol combination therapy represents beneficial properties for minimizing IR-induced arrhythmias during aging; however, in-depth studies are warranted to discover other common anti-arrhythmogenic mechanisms of this combination treatment.

## Acknowledgments

 The authors acknowledge the support of Iran’s National Institute for Medical Research Development (NIMAD, Project No: 957279), Tehran, Iran; Alavi Aging Research Institute, Tabriz University of Medical Sciences, Tabriz, Iran; and Molecular Medicine Research Center, Tabriz University of Medical Sciences, Tabriz, Iran.

## Competing Interests

 The authors declared no potential conflicts of interest with respect to the research, authorship, and/or publication of this article.

## Ethical Approval

 All experimental protocols and procedures were approved by the Ethics Board of Tabriz University of Medical Sciences, Tabriz-Iran (Permission numbers: IR.TBZMED.VCR.REC.1399.283, and IR.TBZMED.VCR.REC.1399.202).
